# N, F Co‐Doped Carbon Derived from Spent Bleaching Earth Waste as Oxygen Electrocatalyst Support

**DOI:** 10.1002/cplu.202400160

**Published:** 2024-10-17

**Authors:** Behzad Aghabarari, Esmat Ebadati, Jesús Cebollada, David Fernández‐Inchusta, María Victoria Martínez‐Huerta

**Affiliations:** ^1^ Department of Nanotechnology and Advanced Materials Materials and Energy Research Center (MERC) Karaj Iran; ^2^ Instituto de Catálisis y Petroleoquímica Consejo Superior de Investigaciones Científicas (CSIC) Marie Curie 2 28049 Madrid Spain

**Keywords:** Electrocatalysis, Spent bleaching earth, Oxygen reduction reaction, Oxygen evolution reaction, Heteroatom doping

## Abstract

Affordable nitrogen and fluorine co‐doped carbon nanostructure was prepared from the hazardous industrial waste of edible oil refinery, spent bleaching earth (SBE), and used as raw material for obtaining high‐performance non‐noble metal bifunctional oxygen electrocatalysts. Waste SBE contains 35 % residue non‐saturated oil as a carbon source and the assistance of montmorillonite (MMT) as the template. This study converts waste SBE into a fluorine‐doped carbon nanostructure through a pyrolysis process followed by removing the aluminosilicate layers of the MMT by HF etching. Furthermore, the impregnation of the support with Co and Fe nitrates readily gives rise to N, F co‐doped carbon (NFC) electrocatalysts, as confirmed by XPS analysis. Electrochemical results evidenced that the Co‐NFC catalyst proved to be a valuable bifunctional competitor for oxygen reduction reaction and oxygen evolution reaction in alkaline media, showing activity in both reactions and superior stability compared with the Fe‐NFC catalyst in accelerated tests. This work offers a straightforward, economical, and eco‐friendly strategy for designing N, F co‐doped carbon‐based electrocatalysts for oxygen reactions in electrochemical devices.

## Graphical abstract

N and F co‐doped carbon nanostructure (NFC) was prepared from the hazardous industrial waste of edible oil refinery, spent bleaching earth (SBE), through a straightforward and economical strategy. Electrochemical results evidenced that the Co‐NFC catalyst proved to be a valuable bifunctional competitor for oxygen reduction and oxygen evolution reactions in alkaline media.

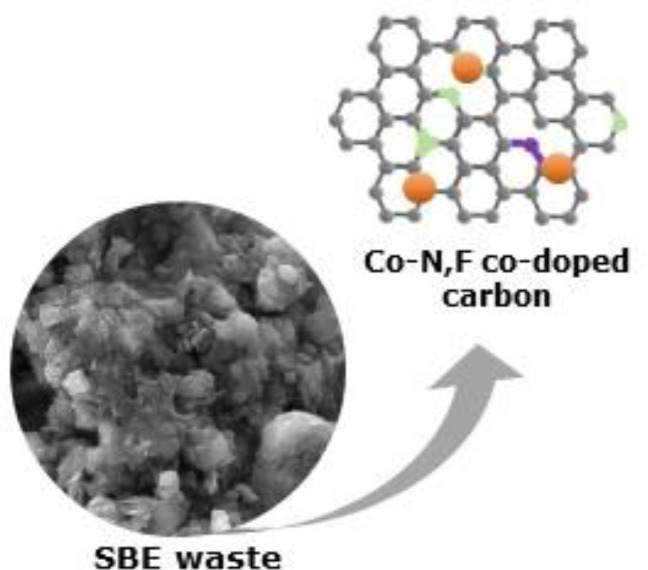

## Introduction

The development of cost‐effective, robust and sustainable bifunctional catalysts based on earth‐abundant elements for the oxygen evolution reaction (OER) and oxygen reduction reaction (ORR) is essential for renewable energy conversions and storage technologies, such as unitized regenerative fuel cells or air batteries.[^1^, ^2^] As the energy requirements of the ORR and OER are different, the bifunctional electrocatalysts of the oxygen electrode will consist of a mixture of an active catalyst for the ORR with an active catalyst for the OER, usually a combination of noble metals such as Pt, Ir, and Ru. Currently, research and development efforts are aiming to replace noble metals in catalysts with non‐noble metals to make catalysts more affordable and improve device durability.[^3^, ^4^] Among different strategies, the combination of advanced carbon structures doped with heteroatoms and transition metals has shown tremendous progress in catalyzing oxygen reactions (ORR/OER).[^5^, ^6^, ^7^, ^8^, ^9^]

Carbon nanostructures N‐doped and co‐doped with various heteroatoms have been widely investigated.[^10^, ^11^, ^12^, ^13^] The heteroatoms are introduced to delocalize carbon matrix electronic clouds and form more efficient active sites. Heteroatom doping increases electrocatalytic activity by enhancing the adsorption and dissociation of oxygen molecules and changing the charge distribution of the bonds between the heteroatom and the carbon atom. Among the various heteroatoms reported, fluorine, with the highest electronegativity, can effectively regulate the electronic structure of the catalyst but also can introduce more abundant defects and other novel effects for favorable electrocatalysis with respect to the other heteroatoms.^[14]^ Fluorine causes the highest charge polarization of adjacent carbon atoms by forming covalent C−F and semi‐ionic C−F moieties and creating a positive charge on the carbon atom. Its control of the electronic structure, conductivity and hydrophilicity can efficiently improve the oxygen electrocatalytic activity of the catalysts.[^14^, ^15^, ^16^, ^17^] Unfortunately, the reported strategies to produce F‐doped carbon have considerable limitations, such as the dangers of F_2_ gas or the high cost of equipment and raw materials.[^17^, ^18^, ^19^] Therefore, it is essential to develop simple and innovative synthesis methods to obtain fluorine‐doped carbon with efficient electrochemical properties.

Using a template is a promising way to synthesize heteroatom‐doped carbon nanomaterials with the desired morphology and structure. Clay minerals are plentiful natural resources that have been used as templates for producing nanostructured carbon materials after the carbonization of carbon sources.[^20^, ^21^] It is necessary to notice that most organic precursors cannot satisfy the demands of the electrocatalytic application. In this regard, industrial waste materials would be candidates to be even more helpful due to their low cost and abundance. Typical waste material from vegetable oil refineries with a global amount of 1.5–2 million tonnes per year is spent bleaching earth (SBE), which usually contains ~20–40 mass% in residual oil. Bleaching earth refers to clays of main montmorillonite (MMT) that can adsorb impurities such as pigments, soap, trace metals, phospholipids, oxidation products and poly aromatics.[^22^, ^23^, ^24^] Because the oil contains high impurities, its extraction has also not been reasonable for food use. It is usually disposed of immediately in a landfill due to the spontaneous ignition of the oil via clay‐catalyzed auto‐oxidation reactions, mainly if the oil is highly unsaturated. On the other hand, fatty materials can leach into the water by this traditional disposal. Recently, by increasing disposal costs of SBE and improving the environmental regulatory rules, recycling of SBE into a useful product via physicochemical methods is regarded as an exciting issue.[^24^, ^25^, ^26^]

This work offers a straightforward and cost‐effective approach to synthesize electrocatalysts based on non‐noble metals and carbon co‐doped with nitrogen and fluorine. The pyrolysis was the waste management option to produce carbon silicate nanohybrid materials from SBE with lower environmental risks. The temperature was controlled to keep the aluminosilicate layers of bleaching earth as the template for converting the residual oil to carbonaceous material. To remove the template and dope fluorine, the nanohybrid material underwent hydrofluoric acid treatment and incorporated non‐noble metal in carbon nanostructure via thermal treatment in an inert atmosphere. Adding Fe and Co using nitrates as precursor salts allows for simple N‐doping of carbon, resulting in co‐doped N and F structures. The effect of transition metals has been investigated in ORR and OER in alkaline solutions.

## Results and Discussion

**Scheme 1:**
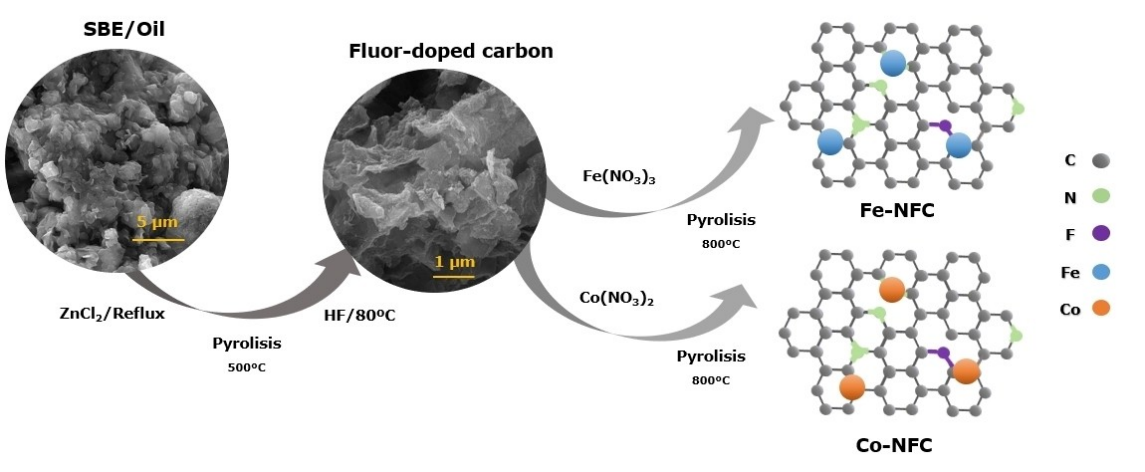
Schematic illustration of the synthetic route for Fe‐NFC and Co‐NFC.

Electrocatalysts were synthesized by depositing Fe and Co nitrate salts on fluoride‐doped carbon (FC) obtained through SBE‐assisted pyrolysis. As schematically shown in Scheme 1, in the first step, the procedure involved preparing carbon silicate hybrid material from SBE and ZnCl_2_, followed by pyrolysis treatment up to 500 °C, which was previously reported by our group.^[25]^ The montmorillonite clay was removed from the hybrid material using hydrofluoric acid (HF) to obtain FC.

In the second step, the monometallic catalysts were prepared by depositing Co(NO_3_)_2_.6H_2_O or Fe(NO_3_)_2_.6H_2_O on FC using the wet impregnation method. The mixture was stirred, dried, and pyrolyzed at 800 °C to obtain the nitrogen and fluoride co‐doped Fe‐NFC and Co‐NFC catalysts.

**Figure 1:**
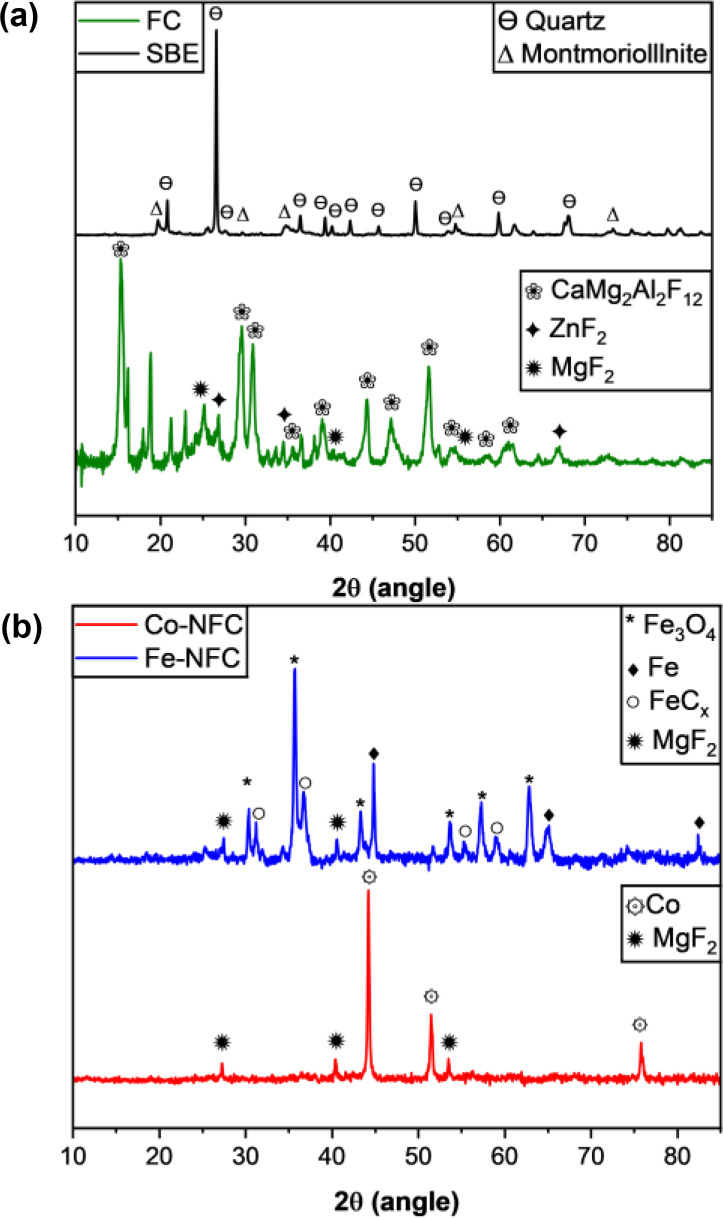
The XRD patterns of (a) SBE and FC, and (b) Fe‐FNC and Co‐FNC.

Figure 1 shows the XRD patterns of SBE and FC (a), and the Fe‐NFC and Co‐NFC electrocatalysts (b). As expected, SBE displays the characteristic diffraction peaks of quartz (JCPDS 46–1045) and aluminosilicate MMT clay (JCPDS 12–0219). The interlamellar spacing of montmorillonite can accommodate an edible oil refinery. With increasing temperature, oil carbonization occurs.^[27]^ Specifically, montmorillonite clay can also contain elements such as calcium (Ca) and magnesium (Mg) incorporated into the crystal structure during its formation.

Therefore, the successive treatments with ZnCl_2,_ pyrolysis at 500 °C and HF likely facilitate the release or rearrangement of these elements, resulting in the formation of several crystal phases such as CaMg_2_Al_2_F_12_ (JCPDS 25–0152), ZnF_2_ (JCPDS 71–0655) and MgF_2_ (JCPDS 72–2231) in the FC sample (Figure 1a). As shown in Figure 1b, the XRD patterns of the Co‐NFC and Fe‐NFC catalysts are markedly different from those of the parent samples. The synthesized samples only contain a few small peaks attributed to MgF_2_, representing the remnants of the FC structure. This indicates that incorporating the metal nitrate salts and the second thermal treatment at a higher temperature (800 °C) eliminated most of the SBE residues. XRD pattern of Fe‐NFC displays characteristic diffraction peaks of Fe_3_O_4_ (JCPDS 99–2215), nonstoichiometric iron carbides phases (JCPDS 06–0686), and metallic iron (JCPDS 34–0529).[^28^, ^29^, ^30^] In the XRD pattern of Co‐NFC, the diffraction peaks correspond to the metallic cubic phase of cobalt (JCPDS 15–0806).^[31]^ Metal loading of Co and Fe was determined by ICP‐OES and is presented in Table S1. Cobalt constitutes 36 wt % and iron constitutes 29 wt %.

**Figure 2:**
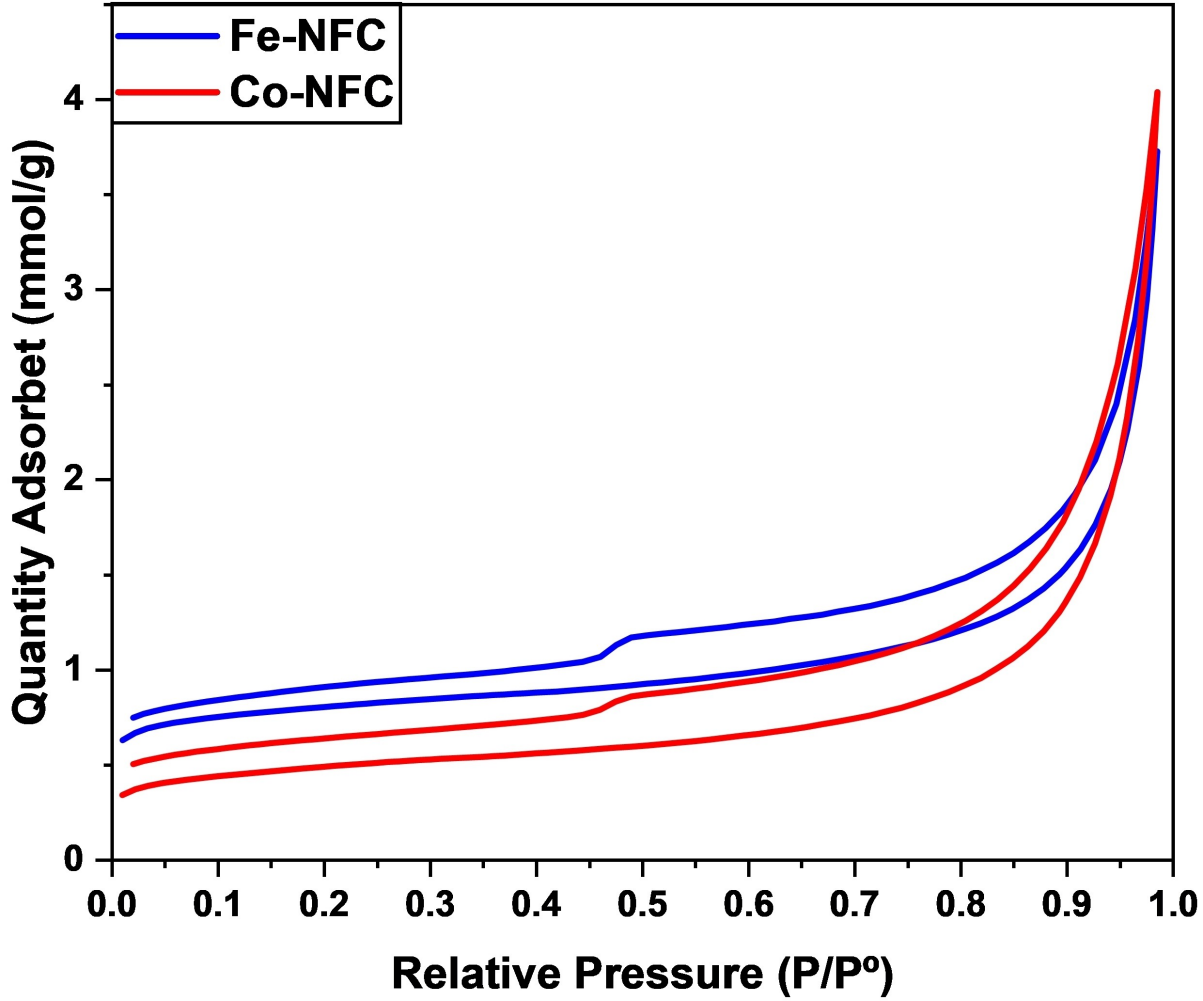
N_2_ adsorption‐desorption isotherms of Fe‐NFC and Co‐NFC.

The N_2_ adsorption‐desorption isotherms and corresponding pore size distribution curves were analysed utilizing the BET method. Table 1 presents a comparative analysis of the structural properties of SBE, FC, Fe‐NFC, and Co‐NFC derived from the BET analysis. The specific surface area and pore size of SBE appear negligible due to pore filling with residual oil. Transformation of residual oil into carbon nanomaterial and subsequent template removal enhanced the specific surface area, developing both micropores and mesopores in the resulting carbon structure of FC. Surprisingly, introducing Co and Fe metals resulted in further augmentation of the specific surface area including the micropores area. This observation supports the notion that incorporating precursor salts facilitates removing remaining residues within FC, even after the HF wash process. Fe‐NFC and Co‐NFC displayed type IV isotherms (Figure 2). The hysteresis loop is characteristic of mesoporous materials. The thermal decomposition of Fe precursor could generate more gas evolution during pyrolysis, which aids in the creation of a more porous structure.

**Table 1:** Summary of the BET data of samples.

Sample	S_BET_ (m^2^ g^−1^)	Micropore Area (m^2^ g^−1^)	V_T_ (cm^3^ g^−1^)	Average pore diameter (nm)
SBE	0.3	–	–	–
FC	26	7	0.107	16
Fe‐NFC	57	32	0.108	7
Co‐NFC	36	12	0.109	12

**Figure 3:**
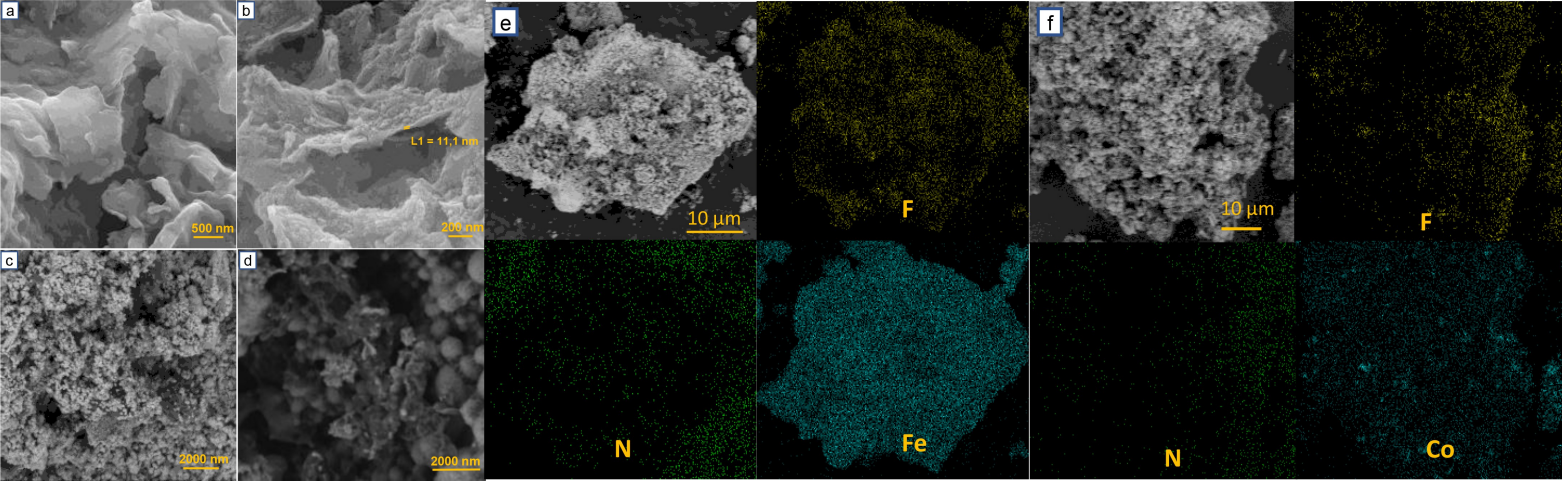
The FE‐SEM images of (a) SBE, (b) FC, (c) Fe‐NFC, (d) Co‐NFC and elemental mapping of (e) Fe‐NFC and (f) Co‐NFC.

The morphologies of the samples were studied by field emission scanning electron microscopy (FE‐SEM) (Figure 3). It can be observed that the surface of the SBE (Figure 3a) is agglomerated and covered by residual oil. The FC surface presents a sheet‐like structure with an increased surface pore (Figure 3b). The morphology change in FC is consistent with BET results. The FE‐SEM and elemental mapping analyses of Fe‐NFC and Co‐NFC are shown in Figure 3(c–f). Both samples contained C, O, Al, Si, F, and a small number of other elements (obtained by EDX, Figure S1).

The amount of fluoride in FC sample was 17.54 wt %. Part of this fluoride was removed from the structure during the second pyrolysis at 800 °C and washing process to obtain values of 2.74 wt % in Co‐NFC and 8.06 wt % in Fe‐NFC. The fluoride can dope the carbon layer during the second pyrolysis and make new inorganic phases such as MgF_2_. Notably, the Si was detected in a small amount, indicating that hydrofluoric acid had successfully removed silicate layers. According to the elemental mapping, the fluorine and nitrogen were incorporated in the Fe‐NFC and Co‐NFC nanostructures and the iron and cobalt, respectively. Elemental analysis was used to determine the nitrogen content. The amount of N was 1.5 wt % and 2.2 wt % for Fe‐NFC and Co‐NFC, respectively. The incorporation of nitrogen is due to metal nitrate salts, which can decompose and react to the carbon at high temperatures. The morphology and structural characterization of FC, Fe‐NFC, and Co‐NFC samples were also examined using transmission electron microscopy (Figure S2). The FC sample exhibited a graphite‐like structure according to graphite‐like FE‐SEM analysis. After adding the metal precursor, the metal nanoparticles were formed in the pyrolysis process and embedded in the matrix of carbon nanostructures. The TEM images of these samples revealed widely variable‐sized nanoparticles ranging from 20 to 80 nm dispersed in the FC matrix.

**Figure 4:**
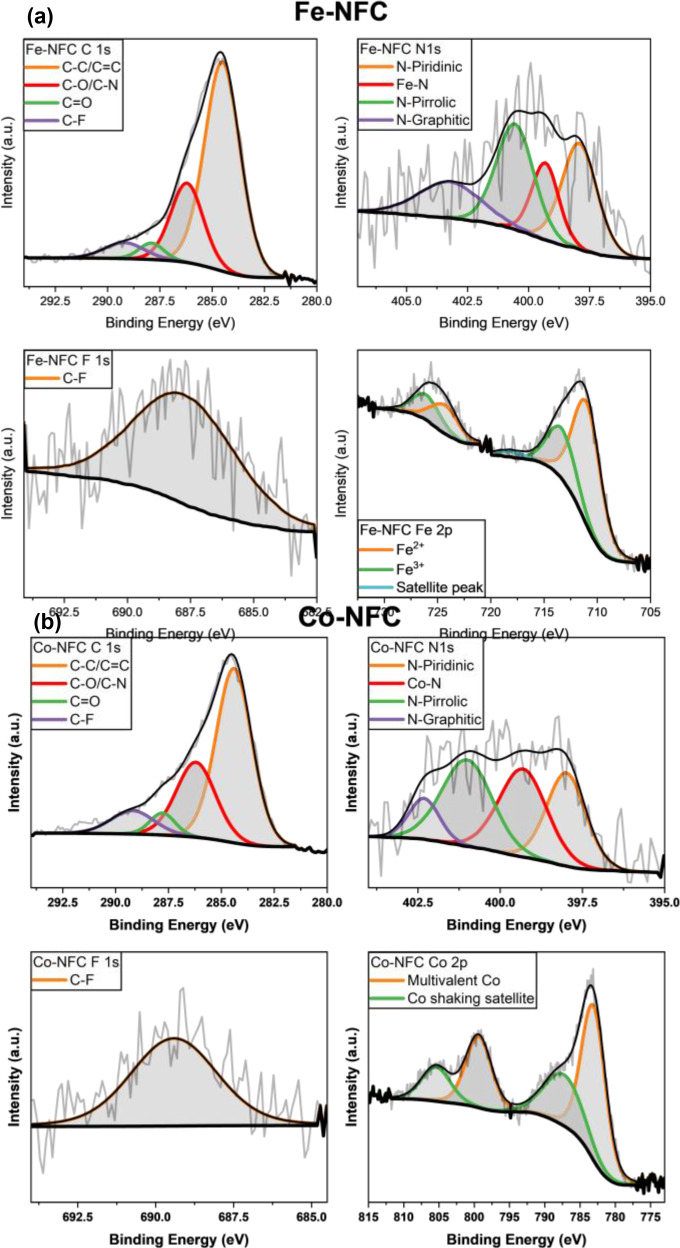
The C 1s, N 1s, F 1s, Fe2p and Co 2p XPS spectra of (a) Co‐NFC and (b) Fe‐NFC.

The surface chemical composition and chemical states of Fe‐NFC and Co‐NFC electrocatalysts were investigated by X‐ray photoelectron spectroscopy (XPS). The deconvoluted XPS spectra for C1s, F1s, N1s, and the corresponding metals (Co2p and Fe2p) are shown in Figure 4a and b. The efficient embedding of F and N atoms into the carbon nanostructure was observed in Fe‐NFC and Co‐NFC XPS spectrums. The high‐resolution XPS C1s spectra of both catalysts were deconvoluted into four peaks around 284.6, 286.2, 287.8 and 289.3 eV, which are ascribed to C−C/C=C, C−O/C−N, C=O and C−F bonds, respectively.[^32^, ^33^] The N1s spectrum of catalysts has been deconvoluted into four subpeaks. The occurrence peaks at ca. 398, 401, and 402.8 eV refer to N bonded to carbon and corresponding to N‐pyridinic, N‐pyrrolic, and N‐graphitic species, respectively.[^34^, ^35^] A peak corresponding to nitrogen coordinated with metal (Fe−N and Co−N) is observed at ca. 399 eV.^[36]^

It is noteworthy that pyridinic nitrogen can increase the density of π‐states in C atoms to the Fermi level and help to fix metal positions with its high chemical stability and decrease the onset potential for ORR by increasing the catalytic current density.^[11]^ On the other hand, the higher electronegativity of the nitrogen atom causes the charge distribution of the nitrogen‐doped carbon atom changes, and thus the carbon atom bonded to the nitrogen atom is positively charged, resulting in enhanced adsorption with oxygen molecules.^[37]^ The F 1s spectra of Fe‐NFC and Co‐NFC electrocatalysts exhibited one peak at 688.8 and 689.4 eV, respectively, associated with semi‐ionic C−F bond.^[38]^ The abundant semi‐ionic C−F bonds are considered beneficial to the conductivity and electrochemical performance of the catalyst.^[39]^ Indeed, high electronegativity F as heteroatom doped into the carbon materials will increase the C atom polarization and cause more surface wettability and oxygen diffusion, significantly improving the catalytic activity of carbon atoms in the FC.[^14^, ^18^] The Co‐NFC catalyst spectrum displays two main peaks at 783.5/788.4 and 799.7/805.7 eV, corresponding to Co 2p3/2 and Co 2p1/2, respectively. The peaks at 788.4 and 805.7 are the shakeup satellite peaks. The peaks fitted at 783.5 and 799.7 eV are attributed to multivalent cobalt originating from surface oxidation of Co. The metallic Co was not observed, probably due to the easy surface oxidation of Co.^[40]^ The binding energies of Co 2p shift towards higher energy, possibly due to the direct bonding of cobalt to nitrogen or carbon of the semi‐ionic C−F bond. The Fe 2p deconvoluted spectra show two doublet peaks, one doublet at 711.6 and 713.5 eV, corresponding to Fe 2p3/2 and the second doublet at 724.7 and 727.4 eV corresponds to Fe 2p1/2 species.^[41]^

**Figure 5:**
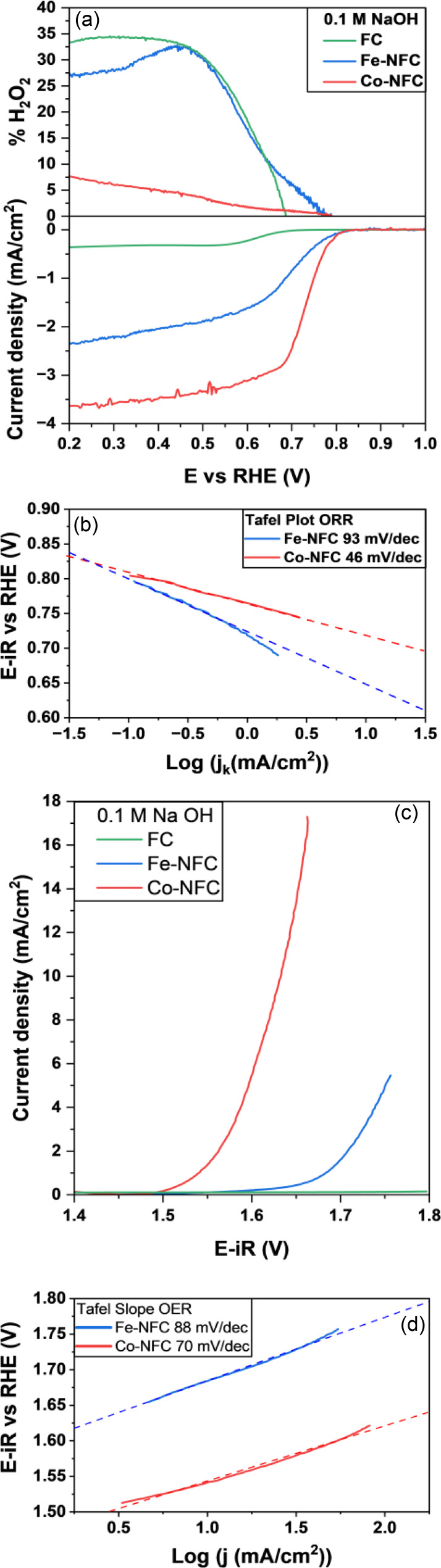
(a) ORR polarization curves (bottom) and hydrogen peroxide yields plots (top) for catalysts, (b) Tafel slopes for ORR obtained from (a), (c) OER polarization curves, (d) Tafel slopes for OER obtained from (c). Scanning rate of 5 mV s^−1^, 1600 rpm in 0.1 M NaOH.

The ORR and OER electrocatalytic activity of Fe‐NFC, Co‐NFC and FC were evaluated using a three‐electrode system with a rotating ring disk electrode (RRDE) in alkaline medium. Figure 5a displays the ORR polarization curves for the catalysts. The top panel shows the Pt ring signal in terms of %H_2_O_2_, which was set to 1.2 V vs RHE. The ORR polarization curve provides relevant electrochemical parameters such as the limit current density, onset potential (E_onset_) and the half‐wave potential (E_1/2_), which are extracted and shown in the bottom panel at the disk signal. It is observed that the ORR activity is strongly influenced by the transition metal, while FC is hardly active. The Co‐NFC with the limit current density of 3.67 mA cm^−2^, E_1/2_ of 0.73 V and E_onset_ at 0.81 V displayed higher activity than Fe‐NFC. Also, it is found that the hydrogen peroxide yield of Co‐NFC is the lowest among the samples, with about 5 % average production. This low yield indicates a four‐electron process, unlike the Fe‐NFC catalyst that has an average production of approximately 30 % of H_2_O_2_. Figure 5c shows Tafel slope values obtained from Figure 5a, bottom. The Tafel slope has been calculated at potential values within the kinetically controlled region of the ORR. It was applied to study the charge transfer behaviours of electrocatalysts, ignoring the surface dynamics and mass transport processes.^[42]^ Co‐NFC and Fe‐NFC present Tafel slope values of 46 and 93 mV dec^−1^, respectively, which means the favourable kinetics of Co‐NFC catalyst in the ORR process.

The OER performances of Fe‐NFC, Co‐NFC and FC have been evaluated in O_2_‐saturated 0.1 M NaOH solution (Figure 5b). OER polarization curves for OER show that the overpotential of Co‐NFC is 400 mV at the current density of 10 mA cm^−2^, which is much lower than that of Fe‐NFC and FC samples. Tafel plots were generated to analyse the OER catalytic kinetics of the catalysts. As shown in Figure 5d, the Tafel slopes were calculated to be 70 and 88 mV dec^−1^ for Co‐NFC and Fe‐NFC, respectively. A smaller Tafel slope indicates a more favourable OER kinetics of Co‐NFC.

**Figure 6:**
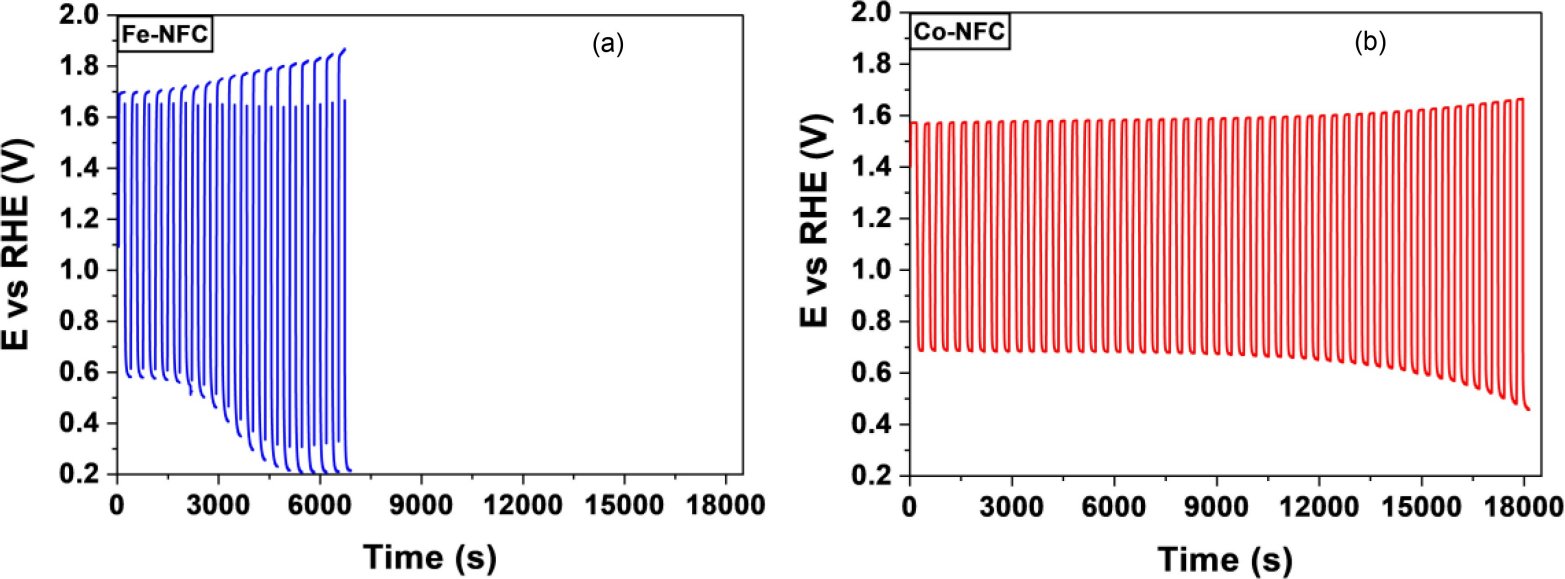
Stability test performed by a chronopotentiometric method, applying cycles of ±5 mA cm^−2^ during 20 s, 0.1 M NaOH. (a) Fe‐NFC and (b) Co‐NFC.

The stability of both Fe‐NFC and Co‐NFC electrocatalysts has been tested using a chronopotentiometric procedure, applying square cycles at constant current density of ±1 mA cm^−2^ for 60 s each until a total of 18000s. A cut‐off value of 1.9 V vs RHE was established. The stability results are showed in Figure 6. The Fe‐NFC catalyst exhibits poor stability behaviour due to its bad OER performance with a cut‐off before 20 cycles. However, Co‐NFC has completed the entire procedure. Thus, the Co‐FNC catalyst has demonstrated exceptional performance in terms of both oxygen reduction reaction and oxygen evolution reaction, while also surpassing the stability of the Fe catalyst.

It has been determined that the iron catalyst presents different crystalline structures such as metallic Fe, Fe_3_O_4_, FeC_x_ including the interaction of Fe species with nitrogen, which can exhibit varying degrees of activity for the ORR and OER. Iron oxides or metallic iron can exhibit some activity for both reactions, but it is typically less efficient compared to other catalyst materials. Even, metallic iron may undergo surface oxidation and passivation in oxygen‐rich environments, which can limit its catalytic activity over time.^[43]^

Surface defects and active sites produced by nitrogen and fluoride on carbon matrix may facilitate ORR and OER processes to some extent, but the overall activity is often lower compared to more specialized catalyst materials. However, iron carbides have been found to possess considerable catalytic activity for oxygen reduction reaction (ORR), while for OER may be more limited. This has been observed particularly when nitrogen or even co‐doped with fluoride is required for the active site formation of FeCx‐based oxygen electrocatalysts.[^36^, ^44^, ^45^, ^46^, ^47^] It appears that the activity of our Fe‐NFC catalyst in electrocatalytic reactions is limited due to the lower presence of carbide or the coverage of other iron species.

The superior activity of Co‐NFC over Fe‐NFC for ORR and OER can be attributed to the unique electronic structure of cobalt. Cobalt can exhibit enhanced activity due to its multiple oxidation states and interaction with nitrogen in a doped‐carbon matrix.[^9^, ^43^, ^48^, ^49^] Moreover, functional groups containing nitrogen, such as graphitic and pyridinic groups, can be highly effective for catalysing the oxygen reduction reaction (ORR)[^9^, ^35^, ^50^] The high activity of Co‐NFC could also be partially attributed to the higher content of semi‐ionic C−F bond, which can be helpful to increase the surface wettability, which is beneficial for oxygen diffusion and the conductivity by the polarization of carbon matrix of the FC. Also, fluorine doping can increase the electron density on the surface facilitating stronger interactions between the catalyst surface and reactant molecules and promoting more efficient adsorption and activation of oxygen species during ORR and OER.[^14^, ^15^, ^51^] Moreover, fluoride doping can help stabilize the active sites on the cobalt catalyst surface, reducing the likelihood of catalyst degradation or deactivation during prolonged electrochemical reactions. This enhanced stability can lead to improved long‐term durability and performance of the catalyst.

## Conclusions

In this work, we demonstrated a straightforward, eco‐friendly strategy to synthesize N, F co‐doped carbon non‐noble metal electrocatalysts. The study successfully synthesized and characterized Fe‐NFC and Co‐NFC electrocatalysts derived from SBE‐assisted pyrolysis. HF etching simultaneously produced the removal of the montmorillonite clay and a fluorine‐doped carbon material. Structural and FE‐SEM analyses revealed the evolution of SBE into fluorine‐doped carbon structures with improved surface area and pore structure. The addition of Fe and Co using nitrates as precursor salts, allows in one‐step N‐doped carbon structures. XPS analysis confirmed the efficient incorporation of F and N and metals into the carbon matrix. Electrochemical testing highlighted the superior performance of Co‐NFC, exhibiting efficient kinetics for both ORR and OER, as well as exceptional stability. Overall, the results underscore the potential of SBE‐derived electrocatalysts for advancing renewable energy technologies.

## Experimental Section

### Material Preparation

The SBE sample was received from the Koorosh food industry (Eshtehard industrial town, Alborz province, Iran). All laboratory chemicals, including ZnCl_2_, HF, Co(NO_3_)_2_.6H_2_O, and Fe(NO_3_)_2_.6H_2_O, were supplied from Merck as analytical grade. Hydrofluoric acid is a highly hazardous substance and necessitates strict adherence to safety protocols such as personal protective equipment. It is handled in a fume hood, and the waste is neutralised with calcium carbonate. Ultrapure water (18.2 MW cm) was used in all the experiments (Milli‐Q, Millipore). In the first step, the carbon silicate nanohybrid material was synthesized according to the procedure reported by our group.^[25]^ A total amount of 100 g of the dried SBE and 45 g of ZnCl_2_ were mixed in 250 ml of deionized water using a magnetic stirrer, heated at 70 °C in a boiler‐reflux condenser for 2 h, and then filtered in a vacuum flask and dried at 105 °C for 24 h. The sample obtained was heated at 150 °C for 30 min under N_2_ atmosphere. Subsequently, the temperature was increased to 500 °C at 5 °C min^−1^ heating rate and held for 2 h to ensure the sample was fully pyrolyzed to carbon silicate nanohybrid material (CSNH). After that, the sample was cooled to room temperature under N_2_ flow. To remove the montmorillonite clay from CSNH, 30 ml of HF was added to 1.3 g of the sample, placed in an ultrasonic bath for 10 min and then in a shaker for 12 h. The sample was rinsed thoroughly with distilled hot water, centrifuged, dried at 80 °C for 24 h and labeled as fluoride‐doped carbon (FC).

The monometallic catalysts were synthetized by depositing Co(NO_3_)_2_.6H_2_O or Fe(NO_3_)_2_.6H_2_O on FC via the wet impregnation method. Typically, FC (1 g) was sonicated in 100 ml of deionized water for 15 min, followed by stirring for 1 h. Then, an appropriate amount of the metal precursor was dissolved in the water and added dropwise to the FC dispersion. The mixture was stirred for 1 h and kept overnight at 70–80 °C followed by dry in an oven at 110 °C for 12 h, and calcined in a furnace at 800 °C. The catalysts were labelled as Co‐NFC and Fe‐NFC.

### Physicochemical Characterization

Elemental analysis was evaluated in a LECO CHNS‐932, and metal loadings of the catalysts were determined by inductively coupled plasma optical emission spectrometry (ICP‐OES) with a Perkin‐Elmer Optima 3300 DV spectrometer. X‐ray diffraction profiles of the powder catalysts were obtained on a PANalytical X’ Pert Pro X‐ray diffractometer with a Cu−Kα source. Transmission electron microscopy (TEM) (CM‐120 Philips) and field emission scanning electron microscopy (FE‐SEM) (TESCAN) were used to determine the morphology of the samples. EDX analysis were carried out with an EDAX Genesis XM2i. The textural properties, such as the specific surface area and pore volume, were obtained by the standard BET method (BELSORP‐mini II, BEL Japan) calculated from pulsed nitrogen adsorption–desorption method at 77 K. The X‐ray photoelectron spectroscopy (XPS) spectra of the catalysts were acquired using an OMICRON ESCA+ spectrometer with a dual x‐ray source (Mg Kα=1253.6 eV, Al Kα=1486.6 eV).

### Electrochemical Measurements

Electrochemical oxygen reduction and evolution tests were carried out employing an AUTOLAB PGSTAT302 N using a three‐electrode system. With a glassy carbon rod as the counter electrode, and a reversible hydrogen electrode (RHE) as the reference electrode. For the working electrode, a rotating ring‐disk electrode (RRDE) was employed, which was composed of a glassy carbon disk with 0.196 cm^2^ of area and a Pt ring. The working electrode was prepared by adding 30 μl of catalytic ink comprising 4 mg of the catalyst in 385 μl of IPA: H_2_O (1 : 1) solution and 15 μl of Nafion® (5 wt %, Sigma‐Aldrich) as a binder. The supporting electrolyte was 0.1 M NaOH aqueous solution. N_2_ (99.99 %, Air Liquid) was employed for all measurements to make an inert environment. The rotate speed of the rotating ring‐disk electrode (RRDE) was controlled by a rotational system (Pine Research Instrumentation, USA). For all measurements, N_2_ (99.99 % Air Liquide) was employed to deoxygenate the electrolyte. The ORR experiments were carried out in O_2_ (99.995 % Air Liquide) saturated alkaline solution at 1600 rpm. The catalysts were submitted to an initial activation process based on 50 cyclic voltammograms (CVs) between 0.05 and 1.2 V vs RHE at a scan rate of 100 mV s^−1^ in deoxygenate supporting electrolyte. The ORR activity was performed by a polarization curve between 1.0 and 0.05 V vs RHE (negative going scan) with a sweep rate of 5 mV s^−1^ v keeping the Pt ring at 1.2 V vs RHE (for quantification of the H_2_O_2_ yield). The percentage of hydrogen peroxide production during the oxygen reduction reaction was calculated according to the following equation:

(1) $$\%H_{2}O_{2}=100\cdot \frac{2\cdot i_{ring}}{Ni_{disk}+i_{ring}}$$

where i_disk_ and i_ring_ are the absolute current from the RRDE disk and ring, respectively, and N is the collection efficiency for Pt ring of the RRDE. The OER activity was performed using a polarization curve between 0.7 and 1.8 V vs RHE (positive going scan) at 5 mV s^−1^ and 1600 rpm. In addition, potential values were iR‐corrected considering the series resistance (48 Ω), which was determined by electrochemical impedance spectroscopy at the open circuit potential and a high frequency (EIS). The durability has been tested using a chronopotentiometric procedure, applying square cycles at constant current density of ±1 mA cm^−2^ for 60 s each until a total of 18000 s. A cut‐off value of 1.9 V vs RHE was established.

## Conflict of Interests

The authors declare no conflict of interest.

1

## Supporting information

As a service to our authors and readers, this journal provides supporting information supplied by the authors. Such materials are peer reviewed and may be re‐organized for online delivery, but are not copy‐edited or typeset. Technical support issues arising from supporting information (other than missing files) should be addressed to the authors.

Supporting Information

## Data Availability

Data sharing is not applicable to this article as no new data were created or analyzed in this study.
