# Supplementary material for: N, F Co‐Doped Carbon Derived from Spent Bleaching Earth Waste as Oxygen Electrocatalyst Support
Source: Chempluschem. 2024 Oct 17;89(12):e202400160. doi: 10.1002/cplu.202400160 (PMC11639644; doi:10.1002/cplu.202400160)
Supplement: Supplementary file 1 — Supporting Information [file CPLU-89-e202400160-s001.pdf]

ChemPlusChem

Supporting Information

N, F Co-Doped Carbon Derived from Spent Bleaching Earth Waste as Oxygen Electrocatalyst Support

Behzad Aghabarari,* Esmat Ebadati, Jesús Cebollada, David Fernández-Inchusta, and María Victoria Martínez-Huerta*

N, F Co-Doped Carbon Derived from Spent Bleaching Earth Waste as Oxygen Electrocatalyst Support

Behzad Aghabarari[a,*], Esmat Ebadati[a], Jesús Cebollada[b], David Fernández-Inchusta[b] and María Victoria Martínez-Huerta[b,*]

[a] Department of Nanotechnology and Advanced Materials, Materials and Energy Research Center (MERC), Karaj, Iran.

[b] Instituto de Catálisis y Petroleoquímica, CSIC, Marie Curie 2, 28049 Madrid, Spain.

*Corresponding authors: b.aghabarari@merc.ac.ir; mmartinez@icp.csic.es

Table S1. Chemical composition of catalysts (Wt %)

**Sample**	**C**	**N**	**Al**	**Fe**	**Mg**	**Co**	**Si**
Fe-NFC	38	1.5	5.3	29	3.2		2.3
Co-NFC	42	2.2	4.7	0.3	3.0	36	1.0
FC	47	0.6	3.9	0.3	2.9		0.6

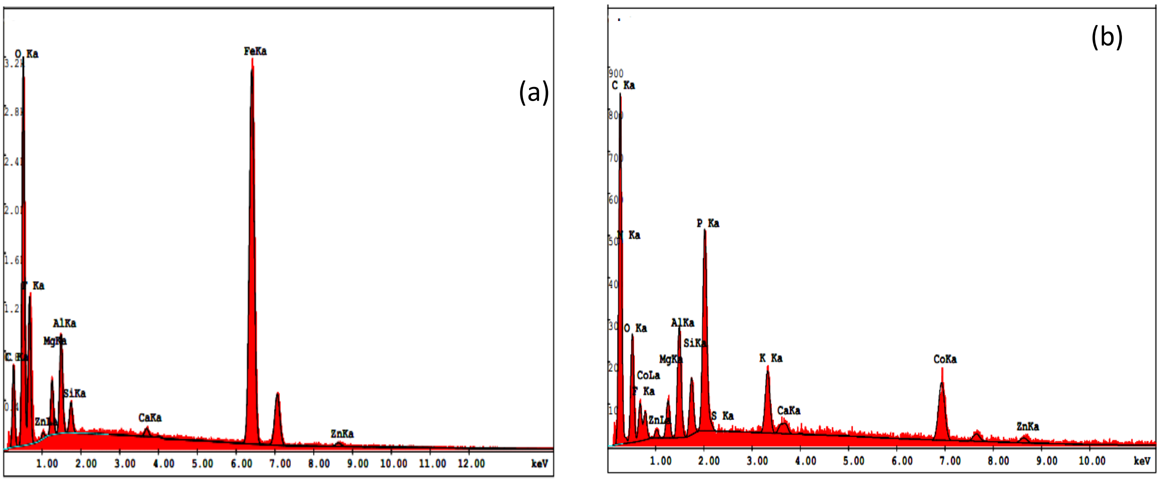

Figure S1. EDX of Fe-NFC (a), Co-NFC (b)

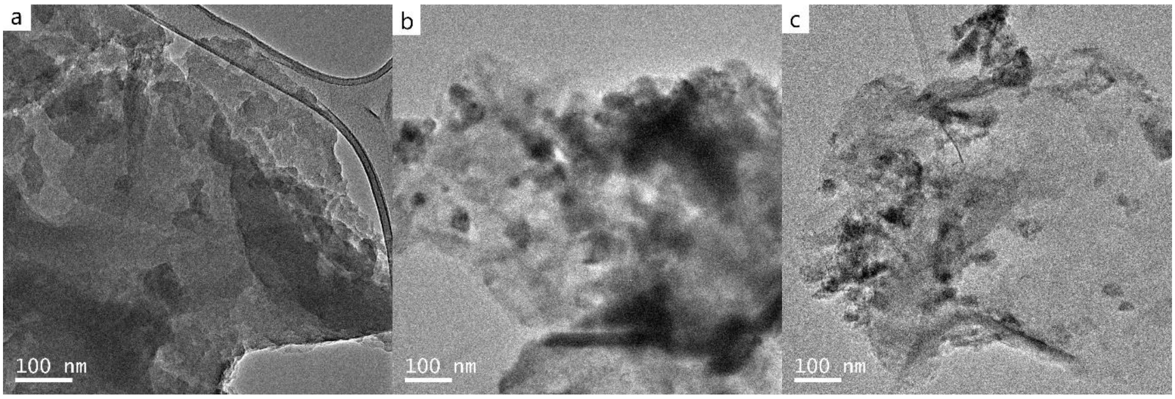

Figure S2. The TEM images of FC (a) Fe-NFC (b), Co-NFC (c)
